# Expression and Characterization of Yeast Derived Chikungunya Virus Like Particles (CHIK-VLPs) and Its Evaluation as a Potential Vaccine Candidate

**DOI:** 10.1371/journal.pntd.0004782

**Published:** 2016-07-11

**Authors:** Shweta Saraswat, T. N. Athmaram, Manmohan Parida, Ankita Agarwal, Amrita Saha, Paban Kumar Dash

**Affiliations:** Virology Division, Defense Research and Development Establishment, Gwalior, India; University of Texas Medical Branch, UNITED STATES

## Abstract

Chikungunya virus (CHIKV) has emerged as a global health concern due to its recent spread in both old and new world. So far, no CHIKV specific drug or vaccine is licensed for human use. In this study, we report production of Chikungunya virus like particles (CHIK-VLPs) using novel yeast expression system (*Pichia pastoris*) and its evaluation as vaccine candidate. The gene encoding structural polyprotein of CHIKV from a recent epidemic strain was cloned into yeast expression system. The multicopy integrants were processed for expression of CHIK-VLPs. The VLPs were purified and confirmed through electron microscopic analysis for their morphological identity with CHIKV. The *in vitro* and *in vivo* evaluation of CHIK-VLPs as vaccine candidate was determined in Balb/c mice. Induction of both humoral and cellular immune response was observed with different doses of CHIK-VLPs. The humoral immune response was studied through different techniques like enzyme linked immunosorbent assay, IgG Isotyping and plaque reduction neutralization test. CHIK-VLPs were found to elicit high titer of antibodies that are able to recognize native CHIKV. Higher level of IgG2a and IgG1 subtypes was identified suggestive of balanced Th1/Th2 response. Both *in vitro* and *in vivo* neutralization activity of CHIK-VLPs antibodies was observed even with low concentration, which shows its high specificity and neutralizing activity against two different CHIKV strains. Neonatal mice receiving anti-CHIK-VLPs antibodies were protected from CHIKV challenge. Induction of cellular immune response was confirmed through higher level of TNF-α, IL-10 and substantial level of IL-2, IL-4 and IFN-γ indicating a balanced response. This is the first report, where CHIK-VLPs has been expressed by *Pichia pastoris* and evaluated for neutralizing activity against CHIKV. These promising results indicate the utility of CHIK-VLPs as a promising vaccine candidate against emerging CHIKV.

## Author Summary

Chikungunya virus (CHIKV) has emerged in many parts of tropics in last decade. The absence of an approved vaccine or antiviral drug for CHIKV makes it one of the important public health challenges. Though attempt to develop a CHIKV vaccine was initiated in 1980s, however it has not succeeded so far. The Virus like particles (VLPs) are now explored as promising vaccine candidate against many viruses viz. HBV, HPV etc. In this study, we report the production of CHIK-VLPs using novel yeast expression system (*Pichia pastoris*) and its evaluation as vaccine candidate. These CHIK-VLPs share morphological identity to native CHIKV. The results indicate that CHIK-VLPs induced both cell mediated as well as humoral response in a balanced manner, which fulfils its criteria as a potent immunogen. Further, antibodies generated against CHIK-VLPs demonstrated efficient *in vitro* and *in vivo* neutralization activity, as evaluated through plaque reduction in Vero cells and protection in CHIKV infected neonatal mice respectively using two different CHIKV strains, which makes it a promising vaccine candidate. The yeast expressed CHIK-VLPs has high potential for development of an effective vaccine candidate against CHIKV during epidemic situations.

## Introduction

Chikungunya virus (CHIKV) is an emerging mosquito transmitted arbovirus of immense public health significance. It belongs to family *Togaviridae* and genus *Alphavirus*. The word Chikungunya comes from Makonde language, meaning “which bends up”, relating to the patients having contoured posture [1]. After its first isolation from Tanzania in 1952, CHIKV has caused numerous outbreaks, both in Africa and Asia. In the last decade, it reemerged in Kenya in 2004, from where virus spread to Indian Ocean islands, Asia, Africa and Europe. Recently it was also reported from Caribbean islands and many parts of Americas [2, 3].

CHIKV is a spherical, enveloped, positive-strand RNA virus of about 60–70 nm. The RNA is enclosed in a ~ 40 nm nucleocapsid that is enveloped by a host derived lipid bilayer supporting viral trimeric glycoprotein-spikes, CHIKV genome is 11,805 nucleotides long. It contains two open reading frames (ORF) encoding the non-structural polyprotein (nsP1-nsP4) and the structural proteins (capsid, envelope proteins E3, E2, 6K, E1) [4, 5].

CHIK-fever caused by CHIKV is a febrile illness associated with rash and severe polyarthralgia that may persist for years. Polyarthralgia, particularly of small joints are the typical symptoms associated with CHIKV [6, 7]. The mortality rate is low (< 0.5%), but is higher in infants less than 12 months old (about 3%) and elderly (more than 60 years old) with concurrent diseases [2]. CHIKV is transmitted mainly by *Aedes aegypti* and *Ae*. *albopictus* mosquitoes. The vector control measures that can play important role in control of infection, have so far proved unsuccessful [8].

Vaccination has the potential of protecting humans and limiting transmission of CHIKV. Currently there are no licensed antiviral or vaccine available commercially. Since 1960 various efforts has been made to develop a vaccine against CHIKV. These include inactivated vaccine, live-attenuated virus vaccines, DNA vaccines, chimeric virus vaccines, subunit protein vaccines and a virus-like particle (VLP) based vaccines. Among these, majority of vaccine candidates are at preclinical stage and/or phase I trial [9].

Conventional inactivated and live attenuated vaccines have been demonstrated to be immunogenic in humans. However requirement of large quantity of virus and BSL-3 containment for vaccine manufacturing are the main hurdles. Though live-attenuated vaccine elicits effective balanced immune response, however, there is a concern of reversion to virulence [10]. A live-attenuated CHIKV vaccine (TSI-GSD218), developed by U.S. Army showed promising results in phase I and phase II [11, 12] clinical trials, however, it was not pursued further due to some side effect like arthralgia in 8% of volunteers [13]. Formalin inactivated vaccine have also shown promising immunogenicity against CHIKV infection [14]. Later subunit E1 & E2 protein based vaccines were evaluated in mice model that elicited good immune response and protection with different adjuvants [15, 16]. DNA vaccine approach was also pursued with C–E2–E1 construct; however it showed practical limitations due to requirement of multiple booster immunization [9]. Recently a novel IRES based live attenuated CHIKV vaccine showed good titre of neutralizing antibodies and protection in various mice model [17].

The virus like particles (VLPs) are non-infectious, nano sized caged architecture composed of viral structural proteins. The presence of complete structural proteins makes it an excellent antigen that strongly mimics the native virus but lack the viral genome making it a safer vaccine candidate. VLPs based CHIKV vaccine candidate produced in mammalian and insect expression system induced an effective immune response even at lower antigen doses [18, 19]. The efficacy of HEK cell based VLP was successfully demonstrated in human volunteers [20]. Further recombinant baculovirus derived VLP was shown to generate good neutralization antibody and also provided protection in mice model [19]. But these VLPs have some disadvantages; CHIK-VLPs based on mammalian cell expression system are related with higher production cost and lower controllability and productivities. Moreover, in baculovirus insect cell expression system, co-production of enveloped baculovirus particles contaminates the vaccine candidate [21]. Moreover, it is difficult to separate baculovirus particles and VLPs; VLPs produced in this system requires chemical inactivation or several downstream processing steps to remove baculovirus infectivity, which will subsequently increase the production cost [22].

*P*. *pastoris* cells readily grow in suspension cultures, which is a benefit of the yeast expression system. *Pichia* expression system is an attractive alternate platform for production of VLPs due to its several advantages including production of protein in native conformation, cheaper operating costs, simple chemical media and free from virus contamination [23] Further, yeast-expressed VLPs were found to be safe and effective [24]. The first yeast derived Hepatitis B virus VLP vaccine received FDA approval in 1986. Till date, a number of *P*. *Pastoris* derived VLP-based vaccines have been approved by US FDA and commercially available worldwide against hepatitis B virus. In India, VLPs based vaccines against HBSAg, derived from *P*. *pastoris* are commercially available [24]. Other *P*. *pastoris* derived Dengue VLPs [25], HPV-16 VLPs [26] and Norovirus VLPs [27] are shown to be elicit good neutralizing antibody titer.

With these advantages, in the present study, we first time exploited *P*. *pastoris* expression system to develop CHIK-VLPs based vaccine candidate and evaluated its immunogenicity in a mice model.

## Materials and Methods

### Virus and cell line

Chikungunya virus (DRDE07) (GenBank Accession number EU372006) having E1:226V belonging to East Central South African (ECSA) genotype isolated from an infected human during 2007 outbreak in Kerala, India and maintained in Division of Virology, DRDE, Gwalior was used as reference standard in this study. Another CHIKV isolate (DRDE06) (GenBank Acc No. EF210157) having E1:226A was used for comparative neutralization study along with DRDE07. All the live virus experiments were performed at BSL-3 facility, DRDE, Gwalior. Vero cell lines were obtained from National Centre for Cell Science (NCCS) Pune, India and were used for virus maintenance, propagation and titration. Vero cells were maintained in Eagles Minimum essential medium (EMEM), supplemented with 10% (v/v) fetal bovine serum (FBS), in a 5% CO_2_ humidified incubator, at 37°C.

### Animals and ethics statement

Four weeks old Balb/c mice were used for immunization purpose and neonatal Balb/c mice (2 day old) were used for *in vivo* neutralization studies. All the animals were obtained from Animal facility, DRDE, Gwalior. The animal experiments had approval from the Institutional Animal Ethics Committee (IAEC) approved by Defence Research Development & Establishment (DRDE), India vide registration number 37/1999/CPCSEA (Committee for the purpose of control and supervision on experiments on animals), Government of India and adopted by the Institutional Biosafety Committee (IBSC). Animals were maintained in accordance with CPCSEA, Govt. of India. The food and water was provided *ad libitum*. After performing the experiment, all animals were euthanized by anesthesia with CO_2_. The animal studies were conducted in a BSL-3 containment facility, following the standard operating procedures for the facility.

### Molecular reagents, plasmid, *Pichia* strain and growth condition

The DNA polymerase, restriction enzymes, and T4 DNA ligase were procured from Fermentas (USA). Synthetic oligonucleotides and chemicals were procured from Sigma-Aldrich (USA). The yeast transfer vector pPIC9K was from Invitrogen (USA). The *P*. *pastoris* GS115 (Invitrogen, USA) was used as the host strain for expression of the Chikungunya VLPs. *P*. *pastoris* GS115 (Invitrogen, USA) was grown at 28°C in Yeast Extract Peptone Dextrose (YPD) Medium. 2% agar was added to the media for plate culture. Transformants were screened in media supplemented with 500 μg/ml Geneticin (Sigma, USA). E. coli DH5α were used in cloning experiments and were grown at 37°C in LB medium supplemented with 100 μg/ml kanamycin.

### Construction of yeast expression cassette having complete CHIKV structural polyprotein gene

RNA from Chikungunya virus (DRDE07) was isolated from infected Vero cell supernatant employing a QIAamp viral RNA kit (Hilden, Germany) according to manufacturer’s protocols. The full length gene encoding CHIKV structural polyprotein was amplified as two fragments known as left and right fragments from CHIKV RNA through conventional RT-PCR. For amplification of left and right fragment, primer set having CHIKYVLPFwd: 5’ TA**TACGTA**ATG GAG TTC ATC CCA ACC C 3’ (*SnaB*I) and CHIKVLPMRev: 5’ CAC GTG AC**C TCG AG**C CCT TCA 3’ (*Xho*I Natural site) CHIKVLPMFwd: 5’ TGA AGG G**CT CGA G**GT CAC GTG 3’ (*Xho*I Natural site) and CHIKYVLPRev: 5’ TA **CCTAGGTTA** TTA TTC TTA GTG CCT GCT G 3’ (*Avr*II) were used respectively. The amplification was carried by using Enhanced avian HS RT-PCR kit (Sigma, USA). The PCR reaction consisted of 5μl of 10X PCR buffer, 3 μl of 25 mM MgCl_2_, 1 μl each of RNase inhibitor, dNTP mix, eAMV-RT, Fwd and Rev Primers (20 pmol) and AccuTaq LA DNA polymerase, 31 μl Nuclease free water and 5 μl of CHIKV RNA. The PCR thermal conditions used were: 48°C for 45 min, 95°C for 5 min, 94°C for 45 sec, 63°C for 45 sec, 72°C for 2 min, for 40 cycles, and finally 72°C for 10 min. The amplified left and right fragment of Chikungunya polyprotein gene corresponded to 1952 bp and 1795 bp respectively. Since both amplified products naming left and right fragment having *Xho*I natural restriction site, so for constructing full length of CHIKV polyprotein gene, both amplified products were digested by using *AvrII* and *XhoI* & *SnaB*I and *XhoI* restriction enzymes. After restriction digestion, both the digested products were ligated at *XhoI* natural site. Full CHIKV structural polyprotein gene, encoding 3747 bp was cloned into pPIC9K yeast transfer vector (Invitrogen, USA) at *AvrII* and *SnaB*I restriction sites. This full length structural polyprotein gene of CHIKV was fused with α-mating factor secretion signal of *Saccharomyces cerevisiae* under control of the methanol-inducible *P*. *pastoris* alcohol oxidase 1 (AOX1) promoter in pPIC9K yeast transfer vector. The resulting pPIC9K-CHIKV-C-E3-E2-6K-E1 DNA was transformed into *E*. *coli* DH5α strain (Invitrogen, USA). The recombinant transformants were selected on Luria-Bertani agar (Himedia, India) supplemented with 100 ug/ml of Kanamycin. Positive recombinant transformants were confirmed by restriction analysis of plasmid DNA using *AvrII* and *SnaB*I and PCR using CHIKYVLP Fwd and CHIKYVLP Rev primers. This was then further confirmed through nucleotide sequencing using ABI 3130 automated DNA sequencer (Applied Biosystems, USA).

### Integration of pPIC9K-CHIKV-C-E3-E2-6K-E1 DNA into *Pichia pastoris* genome and screening of positive transformants

The yeast expression cassette (pPIC9K-CHIKV-C-E3-E2-6K-E1) plasmid was integrated at His4 locus in GS115 strain of *P*. *pastoris* by electroporation as described by manufacture. Briefly, the recombinant plasmid pPIC9K-CHIKV-C-E3-E2-6K-E1 DNA was linearized by *SacI* and 10 μg of the linear DNA has been transformed in to freshly prepared *P*. *pastoris* cells via electroporation using Gene Pulser XCell electroporator (Bio-Rad laboratories, Inc USA.) at 1800V, 20μF capacitance and 200Ω resistance. The transformed cells were plated on RD-His plates (1.34% yeast nitrogen base, 2% dextrose, 0.01% complete amino acid mix apart Histidine, 1 M sorbitol supplement, and 2% agar), and incubated at 28°C for 48 hrs. The colonies obtained were streaked on fresh YPD plates having different concentration of Geneticin (Sigma, USA) *viz* 500 μg/ml, 750 μg/ml and 1000 μg/ ml, Integration of CHIKV structural polyprotein gene at His4 locus of *Pichia* chromosome was verified by amplification with AOX forward and CHIKYVLP Rev primers from genomic DNA of transformants.

### Expression of CHIK VLPs in *P*. *pastoris*

Protein expression was done as described previously with some modifications [28]. Verified PCR positive His^+^ Mut^+^ *Pichia* clone was selected for methanol induction. The glycerol stock of recombinant *Pichia* clone was inoculated into 10 ml YPD medium containing 500 μg/ml Geneticin and was incubated at 28°C in a shaker incubator at 200 rpm until the culture reached an A_600_ of 2–3. Before induction of protein expression, the cells were centrifuged at 2500 × g for 10 min at room temperature. The dextrose containing YPD medium was then replenished with fresh YPM induction media (1% Yeast extract, 2% bacto peptone and 2% Methanol) as to get an A_600_ of 3. Incubation was continued at 28°C on shaker incubator (200 rpm) for 48 hrs. Required volume of methanol was added to flask at every 24 hours interval to sustain induction. Supernatant collected both from un-induced and induced cultures were concentrated using cellulose membrane 10 KD pore diameter (Millipore Corporation, USA) by centrifuging at 5000 rpm for 30 min at 4°C. The samples were stored at -80°C until the expression of CHIK-VLP was analyzed on 10% SDS-PAGE as described by Laemmli [29]. The expressed CHIK-VLPs was confirmed through western blotting using an in-house rabbit anti-CHIKV E2 antibodies and goat anti-rabbit IgG HRP conjugate (Sigma, USA). The colour development was done using H_2_O_2_/DAB substrate/chromogen (Sigma, USA).

### Purification of yeast derived CHIK VLPs by ultra-centrifugation

The CHIK-VLPs in the supernatant were purified by ultracentrifugation through a discontinuous sucrose gradient as described previously [30]. Briefly, 60% and 20% (w/v) sucrose solution was prepared in TNE buffer containing 50 mM Tris-HCl, pH 7.2, 1 mM EDTA, 100 mM NaCl. The discontinuous sucrose gradient was carefully prepared and concentrated VLPs was layered over it and then centrifuged using a TH-641 swinging bucket rotor of ultracentrifuge (Sorvall, USA) for 1 h at 1,00,000 rpm at 4°C. Different fractions of sucrose gradient and 20–60% interface were harvested separately. The collected fractions were then separately analyzed by SDS-PAGE and western blot analysis for the presence of CHIKV structural polyprotein.

### Transmission electron microscopy (TEM)

The purified CHIK-VLPs and inactivated CHIKV were fixed in 4% formaldehyde and negatively stained with 1.5% phosphotungstic acid (PTA), pH 7.2. Briefly, 1.0 μl of the VLPs and inactivated CHIKV were placed onto a carbon coated Formvar-filmed copper grid (TAAB, UK) and CHIK-VLPs and inactivated CHIKV were allowed to attach to the surface for 1 min. The grid was washed thrice in sterile triple distilled water by floating the grid on water droplets for 45 seconds to remove excess sample and sucrose. Finally the samples were negatively stained by 1.5% PTA solution as described previously [30]. The air dried grid was examined using Transmission electron microscope.

### Mice immunization

The immunogenicity of CHIK-VLPs as vaccine candidate against CHIKV was evaluated in 4 week old female Balb/c mice. For the preparation of immunogen, the purified CHIK-VLPs were mixed with equal volume of Freund’s adjuvant (Sigma, USA). Four groups (n = 10 each) were immunized subcutaneously with 10 μg, 20 μg and 40 μg of yeast derived CHIK-VLPs in Freund’s adjuvant and a control group was immunized with PBS. To determine humoral and cell mediated response all groups were boosted with same formulation on 14, and 28 day after first immunization. Mice immunization was also done with inactivated CHIKV to compare humoral response.

### Evaluation of humoral immune response

#### Determination of antibody titer through ELISA

The anti-CHIK-VLPs IgG response against CHIK-VLPs was determined through indirect ELISA at 14, 28, 42, 56 and 140 days of post-vaccination. Briefly, 96-well ELISA plate (Nunc, USA) was coated with CHIK-VLPs (300 ng/well) followed by blocking with 3% BSA (Sigma, USA) on next day. The plate was then washed five times with PBST, followed by incubation with two fold serially diluted post-vaccinated sera starting from 1:250 to 1:51200 dilutions in triplicate wells (100 μl/well) including healthy non-vaccinated sera for 1 h at 37°C followed by five washing with PBST. HRP-labeled goat anti-mouse IgG (Sigma, USA) (1:5,000) was added. This was followed by washing (as above) and development with TMB/H_2_O_2_ (Sigma, USA) as substrate chromogen. Finally colour development was stopped using 1N H_2_SO_4_ and plate was read at 450 nm by microplate reader (Biotek Instruments, USA). Cut-off value was calculated as the mean absorbance (+2 SD) from control sera assayed at 1:250 dilutions. The endpoint IgG titers were then calculated as reciprocal of the highest serum dilution giving an absorbance more than the cut-off.

Similar indirect ELISA was performed for evaluating the potential of CHIK-VLPs in recognizing native CHIKV. In this, method 300 ng/well of purified native CHIKV was coated as antigen and the rest procedure remain same.

#### Antibody isotyping

The antibody isotyping of CHIK-VLP immunized sera and inactivated CHIKV sera was determined using mouse antibody subtyping kit (Sigma, USA). Briefly, CHIKV-VLP or inactivated CHIKV coated ELISA plate was incubated with 100μl (1:1000) of immunized sera and incubated for 1 h at 37°C followed by three washing with PBST. Wells were incubated with 100μl (1:1000) of goat anti-mouse IgG specific for each subtype (IgG1, IgG2a, IgG2b, IgG3) (Sigma, USA), at 37°C for 1 h. Following washing, 1:5000 dilution of rabbit anti-goat IgG HRP conjugate (Sigma, USA) was added and incubated at 37°C for 30 min. Following washing, plate was developed using TMB/H_2_O_2_ chromogen and absorbance was measured at 450 nm.

#### Immunofluorescence assay

Sera samples were tested for their ability to recognize the Chikungunya virus by immunofluorescence assay. Vero cells were seeded in a 6 well plate with a cover slip. At 90% confluency, cells were rinsed with PBS and 10^3^ PFU of CHIKV was added to the respective wells. The plate was incubated at 37°C for 1 h, with shaking at 20 min intervals. After adsorption, plate was rinsed once with PBS and then replenished with MEM containing 2% FBS and incubated further at 37°C for 24 hrs. The cells were rinsed thrice with PBS, fixed with chilled methanol for 30 min, followed by blocking with 5% BSA for 2 h at 37°C. The cells were then washed thrice with PBS and permeabilized with 0.1% Triton X-100 for 15 min. The cells were incubated for 1h at 37°C with 1:1000 dilution of hyperimmune sera raised against CHIK-VLP. Similarly dilution of pre-immune sera was used as control. The cells were then washed and incubated with Fluorescein isothiocyanate (FITC)—conjugated goat anti mouse antibody (1:100) (Sigma, USA) for 45 min at room temperature in dark. Cells were then washed and fluorescence was visualized using a Carl-Zeiss Aximot 2 (Olympus IX 71, Germany) microscope.

#### *In vitro* neutralization activity of mice sera immunized with CHIK-VLPs

*In vitro* neutralization activity of mice sera immunized with CHIK–VLPs was carried out using two different techniques *viz* plaque reduction neutralization test (PRNT) and immunofluorescence assay (IFA). Two-fold serial dilutions of heat inactivated sera were prepared in MEM and added to equal volume of the CHIKV containing 10^2^ pfu. The virus control was included without serum. The diluted serum-virus mixtures were incubated at 37°C for one hour. 200 μl/well of serum-virus mixture was then used to infect Vero cells for both PRNT and IFA.

Briefly in PRNT, plate was incubated at 37°C for one hour. The serum-virus mixtures were completely removed and the wells were overlaid with 1ml/well of MEM containing 2% FBS and 1.25% methyl cellulose (Sigma, USA). Plate was incubated at 37°C in 5% CO_2_ for 3 days. Cells were washed with PBS and fixed with chilled methanol for 1 hr. The fixed cells were stained with 0.25% crystal violet. The plaques formed were counted. The neutralizing titre is considered as the highest dilution of the immunized sera that showed more than 50% plaque reduction, compared to virus control (serum negative control).

Briefly in IFA, following 24 hrs infection, immunofluorescence was developed by using anti-CHIK-VLP and FITC conjugated goat anti mouse antibody (1:100) (Sigma, USA) as primary and secondary antibody respectively. Cells were than washed and fluorescence was visualized using a Carl-Zeiss Aximot 2 (Olympus IX 71, Germany) microscope. The highest dilution of immunized sera that showed reduction in fluorescence as compared to virus control was considered as neutralization titer.

#### *In vivo* neutralization test

*In vivo* neutralization activity of immunized CHIK-VLP mice sera, was determined via passive immunization of purified IgG intraperitoneally as descried previously [31, 32]. Total IgG was purified from post-immunized sera (42 days post immunization) by using protein-A column (Sigma, USA). 10 μg of purified IgG raised against yeast derived CHIK-VLPs was immunized to suckling mice (2 days old, n = 10) via intraperitoneal route. For control set, same amount of non-specific IgG from pre-immune sera was used. After 48 hrs of IgG immunization, different groups of suckling mice were subcutaneously inoculated with 10^6^ PFU of CHIKV. Symptoms were recorded at regular interval of 24 h up to 10 days of post-infection. Gain in body weight, percentage survival and serum viremia were determined. On day 3 and 6 after CHIKV inoculation, 3 mice pups in each experimental and control group were bled to determine serum viremia via qRT-PCR as described earlier [33].

### Assessment of cellular immune response

Cellular immune response against CHIK-VLPs was determined by cytokine profiling. The expression level of different Th1 and Th2 cytokines i.e. TNF-α, IFN-γ, IL-2, IL-4, IL-6 and IL-10 from the culture supernatants of splenocytes of CHIK-VLPs immunized mice and mock immunized mice were measured by sandwich ELISA using BD OptEIA Kit, (BD Biosciences, USA).

Briefly, after 7 days of post-booster vaccination, three mice from all the groups were sacrificed and their spleens were retrieved. Splenocyte cell suspension prepared from each mouse (1×10^6^ cells per well) were seeded in a 24-well plate in triplicate. To measure CHIKV specific responses, cells were stimulated with different doses of inactivated CHIKV (10 μg, 20 μg and 40 μg) corresponding to respective mice groups. Concanavalin A (Con A, 5μg/ml; Sigma, USA) as positive control and negative control (without antigen) were also included for each group. The plates were incubated at 37°C in 5% CO_2_ humidified incubator. After *in vitro* stimulation, cell supernatants were collected from the wells at 48 and 72 hrs and stored at -80°C prior to use. The cell supernatant was then centrifuged at 15,000g for 30 minutes at 4°C and assayed for cytokines by BD mouse sandwich ELISA Kit (BD Biosciences, USA). Cytokine expression was determined using standard curve and presented as picograms per millilitre (pg/ml).

### Statistical analysis

All experiments were performed at least thrice in duplicate. Statistical comparisons for antibody titer, IgG isotypes and cytokine levels were done using GraphPad Prism 6 software (La Jolla, CA), by one way ANOVA using Multiple Comparison Procedure (Fisher LSD Method). Statistical analyses of serum viremia and body weight gain were performed using an unpaired *t*-test. *p* < 0.05 was considered to be statistically significant.

## Results

### Structural polyprotein gene of chikungunya virus was successfully transformed into GS115 strain of *Pichia pastoris* as multi copy integrants

The full length structural polyprotein gene encoding capsid, E3, E2, 6K and E1 protein of CHIKV was amplified as left and right fragment. Amplification of left fragment and right fragment by RT-PCR resulted in 1.952 Kbp and 1.795 Kbp amplified product respectively (S1A Fig). Since both fragments had *Xho*I natural restriction site they were ligated by T4 DNA ligase resulting 3.747 Kbp product. The full CHIKV structural polyprotein gene, encoding 3.747 Kbp was transformed into pPIC9K yeast transfer vector (Invitrogen, USA) at *AvrII* and *SnaB*I sites resulting pPIC9K-CHIKV-C-E3-E2-6K-E1 construct. Positive transformants having full CHIKV structural polyprotein gene was confirmed by restriction digestion with *AvrII* and *SnaB*I and PCR amplification with CHIKYVLP forward and CHIKYVLP reverse primers. Correct integration resulting 3.747 Kbp DNA band (S1B & S1C Fig). The nucleotide sequencing further confirmed the absence of any mutation during RT-PCR and cloning. Recombinant pPIC9K-CHIKV-C-E3-E2-6K-E1 expression cassette (S1D Fig) was further integrated into genomic DNA of *Pichia* by electroporation. 49, 32 and 25 colonies were found on YPD plates with 500, 750 and 1000 μg/ml of Geneticin (S2A Fig). Further integration of this gene was confirmed by PCR that revealed an amplicon of 3.9 Kbp (S2B Fig). The intensity of the PCR bands on agarose gel also found corresponding to the concentration of Geneticin. Thus, results were in concordance with the Geneticin sensitivity assay.

### Yeast (*P*. *pastoris*) expressed CHIK-VLPs

**Fig 1:**
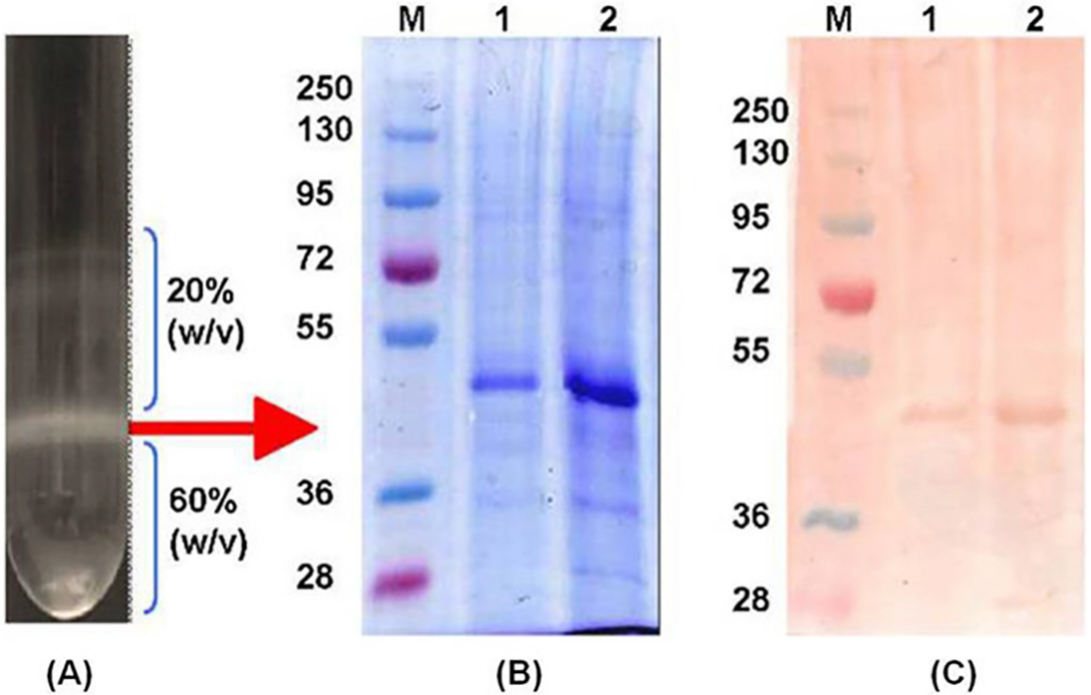
Purification and characterization of CHIK-VLPs. (A) Separated interface fractions of Chikungunya virus like particles in sucrose density gradient centrifugation; (B) SDS-PAGE analysis of purified CHIK-VLPs from discontinuous sucrose gradient; (C) Western blot analysis of purified Chikungunya virus fractions. Lane M: prestained molecular weight marker (Fermentas, USA); Lane 1: Just above interface; Lane 2: At interface (20–60%).

**Fig 2:**
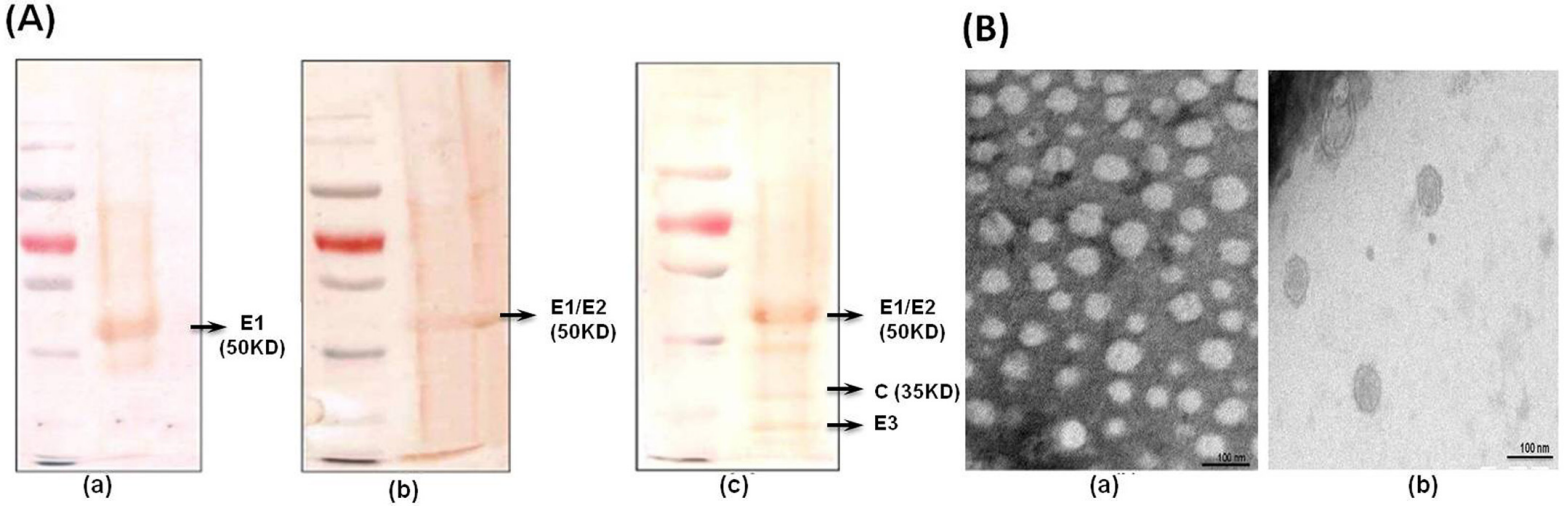
Characterization of VLPs. **(A) Immuno-blotting using different CHIKV specific Ab.** (a) Western blot using anti-E1 pAb; (b) WB using anti-E2 mAb; (c) WB using anti-CHIKV pAb; **(B) Transmission Electron Microscopy analysis of purified CHIK-VLPs and native CHIKV:** (a) Electron micrograph of purified CHIK-VLP at 2, 00,000 X; (b) Electron micrograph of purified native CHIKV at 2, 00,000 X.

Positive clone was selected from *P*. *pastoris* transformed with the pPIC9K-CHIKV-C-E3-E2-6K-E1 plasmid. Western blot of CHIKV proteins in yeast supernatant with anti-CHIK E2 antibodies revealed approximately 50 KD protein band that corresponds to the size of major structural protein of CHIKV (E1 & E2). The concentrated *Pichia* yeast supernatant was purified through 20–60% discontinuous sucrose density gradient centrifugation. SDS-PAGE analysis of various purified fractions revealed localization of majority of CHIK-VLPs at 20–60% sucrose interface (Fig 1A). The E1 and E2 glycoproteins migrate together on the SDS-PAGE gel and formed a single band at ~50 KD (Fig 1B). The Capsid protein formed a distinct band at ~32 KD. Different structural proteins in density gradient fractions were also confirmed through western blotting employing anti-CHIK E2 monoclonal, anti-CHIK E1 and anti-native CHIKV polyclonal antibodies (Fig 2A). The TEM examination revealed the presence of spherical particles of approximately ~65–70 nm. The average diameter of Chikungunya core was approximately 40 nm (Fig 2B.a). TEM analysis of native CHIKV revealed comparable morphological similarity to infectious virus (Fig 2B.b). Finally, the VLPs were dialyzed and concentrated, giving a final protein concentration of 60 μg/ml.

### Determination of humoral immune response

#### ELISA antibody titer (Total IgG titer)

**Fig 3:**
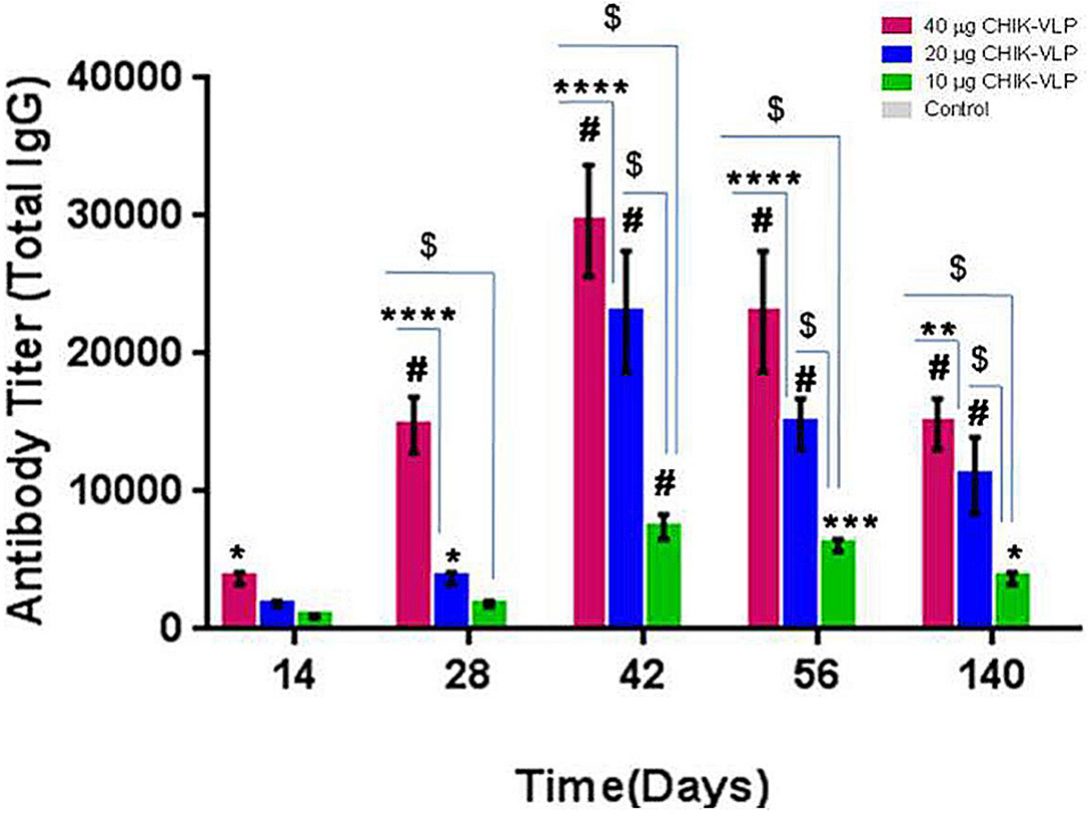
Measurement of serum IgG antibody titers in Balb/C mice immunized with CHIK-VLPs. Sera collected after first booster 14, 28, 42, 56 and 140 days of post-vaccination from immunized groups with 40 μg, 20 μg and 10 μg CHIK-VLPs and antibody titer was measured by indirect ELISA. Data represented as mean antibody titers with S.D. of ten Balb/c mice in each group. Analysis was done by one way ANOVA (Fisher LSD Method). ****P <0.0001(significance with respect to 20 μg CHIK-VLPs); ^$^P < 0.0001; (significance with respect to 10μg CHIK-VLPs); ^#^P < 0.0001; ***P < 0.001; *P < 0.01 (significance with respect to control).

The assay antibody response in vaccinated mice was examined through an indirect ELISA using CHIK-VLPs. The highest IgG endpoint titer to CHIK-VLPs was 4 × 10^3^ in sera of 40 μg CHIK-VLPs group whereas it was 2 × 10^3^ in 20 μg CHIK-VLPs and 1 × 10^3^ was in 10 μg CHIK-VLPs group at 14 days post immunization. The highest IgG endpoint titer after first booster (28 days) was 1.6 × 10^4^ in 40 μg CHIK-VLPs group whereas 4 × 10^3^ in 20 μg CHIK-VLPs and only 2 × 10^3^ in 10 μg CHIK-VLPs. The peak ELISA antibody titer was obtained on 42 days post-vaccination After second booster (42 days) antibody titer were 3.2 × 10^4^, 2.56 × 10^4^ and 8 × 10^3^ in mice immunized with 40 μg, 20 μg and 10 μg CHIK-VLPs respectively. Serum anti-CHIK-VLPs antibody levels in immunized group was found significantly higher in all of three concentrations compared to controls on 42 day (^#^P < 0.0001), whereas on 56 and 140 days was significantly higher in 40 μg and 20 μg concentration with respect to control mice sera (^#^P < 0.0001) and the same pattern persisted up to 140 days post vaccination. Titre of serum antibody increased in dose dependent manner. ELISA titers were also substantially increased following booster doses. The antibody titer in 40 μg CHIK-VLPs was found to significantly higher than 20 μg CHIK-VLPs (****P < 0.0001) and 10 μg CHIK-VLPs (^$^P < 0.0001) (Fig 3).

#### IgG subtyping demonstrate high levels of IgG1 and IgG2a

**Fig 4:**
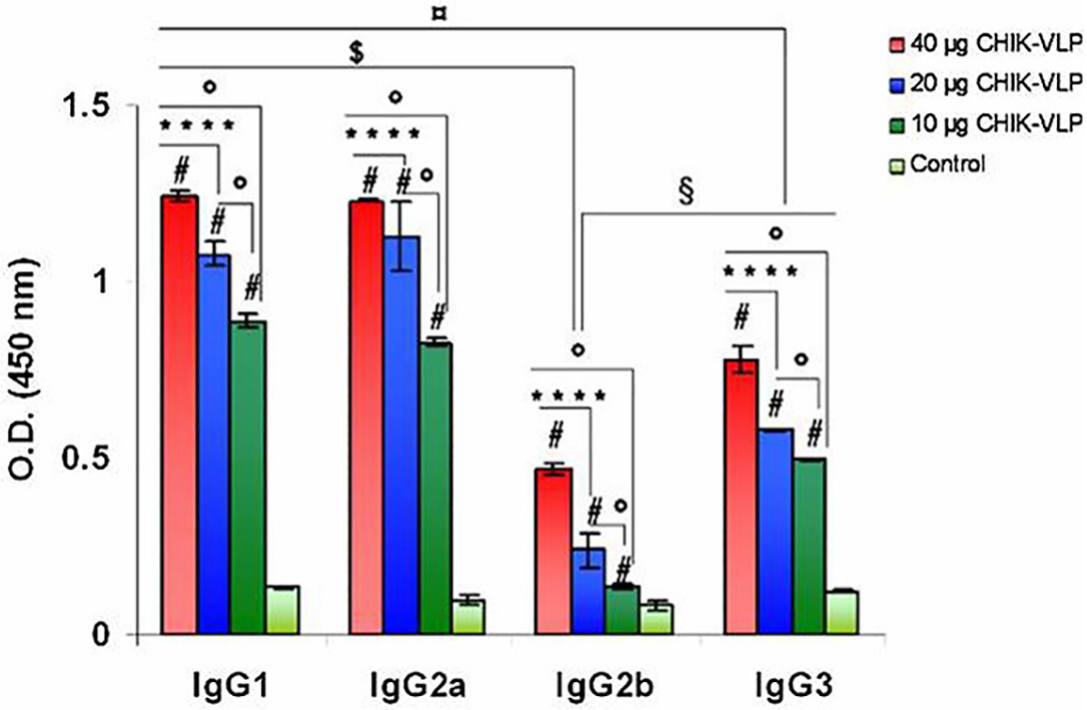
Measurement of serum IgG isotypes titers in immunized BALB/c mice. Profile of IgG isotypes in sera from immunized animal groups (40 μg, 20 μg and 10 μg CHIK-VLPs). Data represented as mean antibody titers with S.D. of ten Balb/c mice in each group Analysis was done by one way ANOVA, (Fisher LSD) ^**#**^P < 0.0001(significance with respect to control); ****P < 0.0001(significance with respect to 20 μg CHIK-VLPs); °P < 0.0001(significance with respect to 10 μg CHIK-VLPs); ^**$**^P < 0.0001(significance with respect to IgG2b); ^§^P < 0.001(significance with respect to IgG2b); ^**¤**^P < 0.0001(significance with respect to IgG3).

Due to the strong antibody response, isotyping analysis was performed in the pooled sera samples to determine the predominant isotypes of antibodies produced in mice in response to vaccination. Significantly (**#**P < 0.0001) high level of all four isotypes IgG1, IgG2a, IgG2b and IgG3 were observed in all groups (40 μg, 20 μg and 10 μg CHIK-VLPs) compared to control group. Among the IgG subclasses, IgG1 and IgG2a appeared to have the highest and approximately same levels, although IgG3 levels were also high but IgG2b levels were the lowest. Immunization of different doses of CHIK-VLPs with FCA showed the balanced immune response with approximately high and same amount of IgG1 and IgG2a. There is no significant difference with the level of IgG1 and IgG2a, whereas with respect to IgG2b, the level of IgG1 and IgG2a were found to significantly (^**$**^P < 0.0001) higher. The level of IgG3 was found to be significantly lower (^¤^P < 0.0001) than IgG1 and IgG2a in all doses of CHIK-VLPs (Fig 4). A comparison of the magnitude of CHIKV antibody response was made between VLPs and inactivated CHIKV, in which antibody subtype profile was comparable between CHIK-VLPs and inactivated CHIKV immunized mice (S3 Fig).

#### Yeast derived CHIK-VLPs generated high titer of neutralizing antibodies that recognized native CHIKV

**Fig 5:**
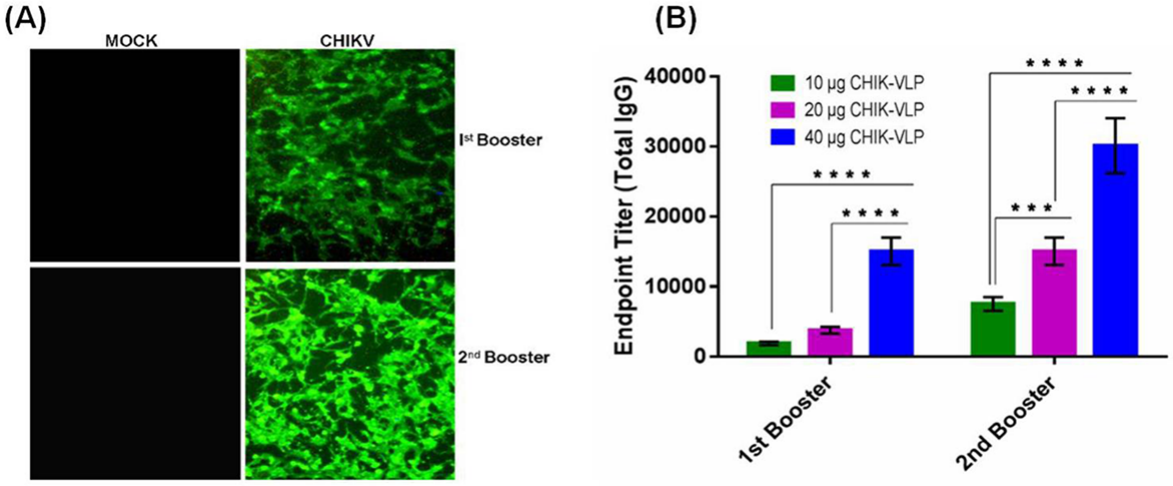
Antibody raised against CHIK-VLPs was evaluated for their specificity against Chikungunya virus. (A) Determination of immunofluorescence against Chikungunya virus by using sera of different booster of 40 μg CHIK-VLPs; (B) Antibody titration of both booster of all groups against Chikungunya. Data represented as mean antibody titers with S.D. of ten Balb/c mice in each group Analysis was done by one way ANOVA, (Fisher LSD) ****P < 0.0001, ***P < 0.001.

To evaluate whether anti CHIK VLPs are able to recognize native Chikungunya virus or not, immunofluorescence assay and indirect ELISA had been performed. 1^st^ and 2^nd^ booster sera raised against 40 μg CHIK-VLPs were analyzed through immunofluorescence assay in CHIKV infected Vero cells. Both booster sera samples containing antibodies recognized CHIKV in infected cells (Fig 5A). Compared to 1^st^ booster, higher level of CHIKV antigen was recognized by anti-CHIK-VLPs of 2^nd^ booster. Serum of mock immunized mice did not show any fluorescence. Thus, mice sera of both the boosters of different groups were found to recognize native CHIKV with high endpoint titer. As seen in Fig 5B a dose dependent antibody titer has been seen with significant difference in antibody titer of all three doses of both boosters (****P < 0.0001).

#### *In vitro* virus neutralization activity of mice sera immunized with CHIK-VLPs via PRNT

**Fig 6:**
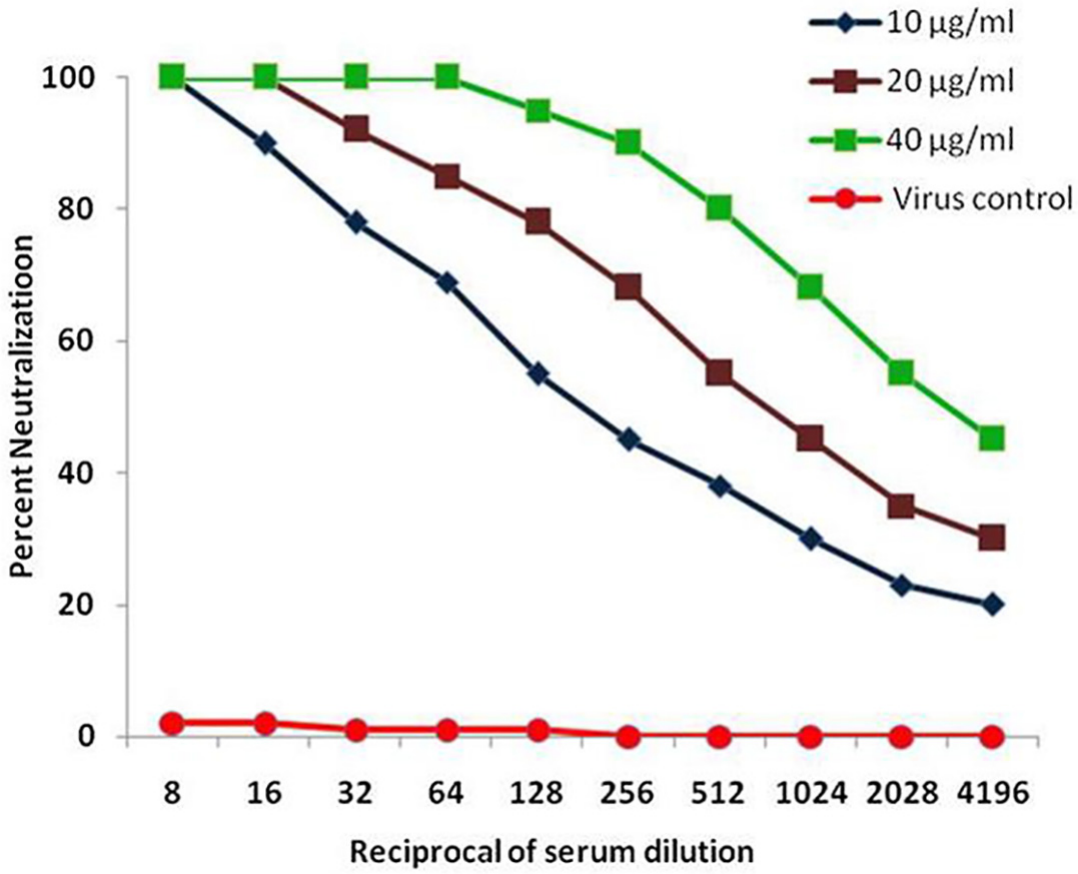
*In vitro* virus neutralization activity of mice sera immunized with CHIK-VLPs. *In vitro* neutralization activity of mice sera immunized against CHIK-VLP was evaluated against two different CHIKV strains (DRDE 07 and DRDE 06). Serial two fold dilution of mice sera starting from 1:8 to 1:4196 were used to neutralize 10^2^ pfu virus (DRDE 07 and DRDE 06). The PRNT_50_ titer of mice sera were 1:2048, 1:512 and 1:128 for 40 μg CHIK-VLPs, 20 μg CHIK-VLPs and 10 μg CHIK-VLPs respectively.

The *in vitro* neutralization activity of mice sera immunized against CHIK-VLP was checked against two different strains of CHIKV. The PRNT_50_ titer of mice sera against both the strains (DRDE 07 and DRDE 06) were 1:2048, 1:512 and 1:128 for 40 μg CHIK-VLPs, 20 μg CHIK-VLPs and 10 μg CHIK-VLPs respectively (Fig 6).

#### Immunized mice showed full protection and survival from CHIKV

**Fig 7:**
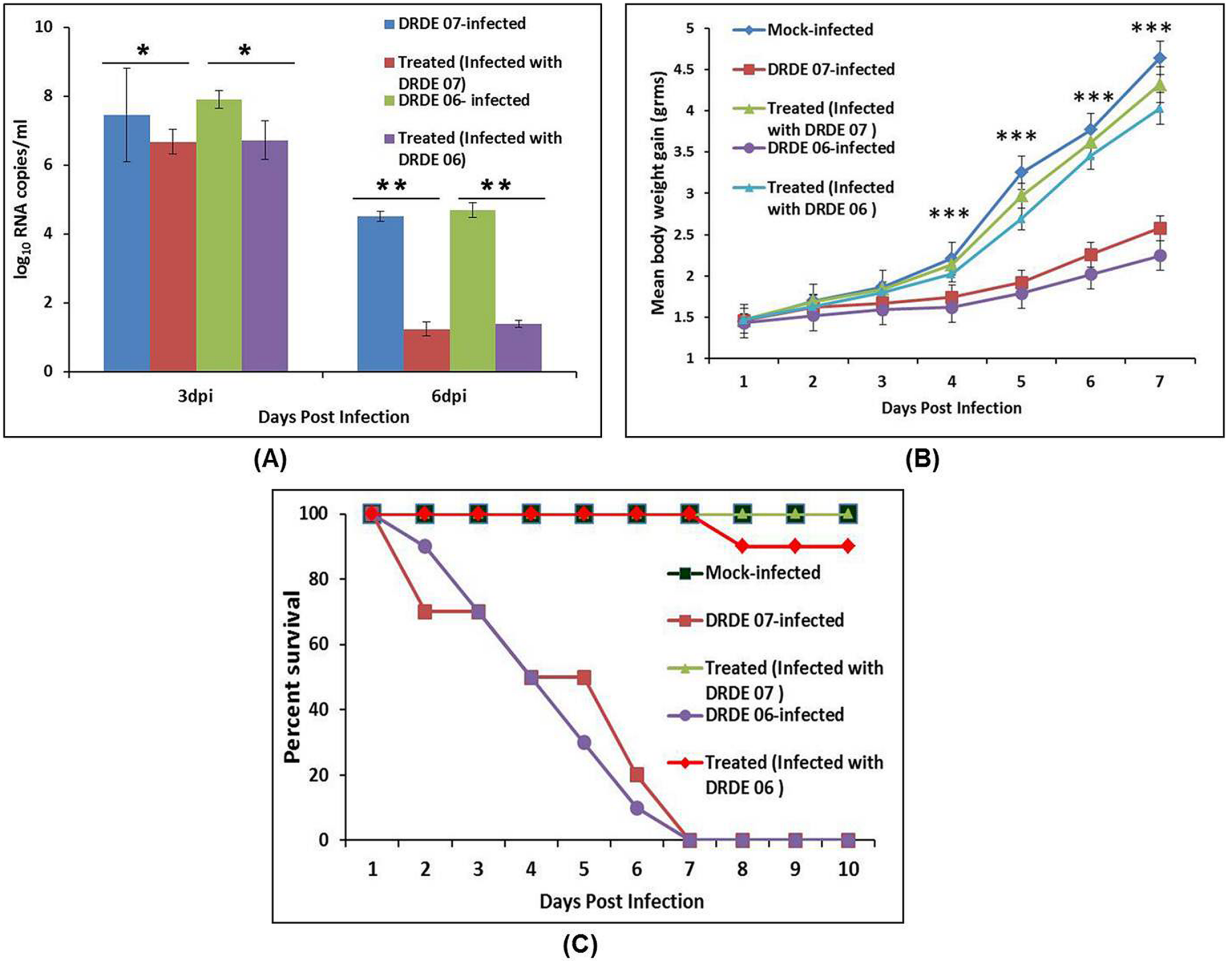
*In vivo* virus neutralization activity of mice sera immunized with CHIK-VLPs. (A) Percentage survival of the all the mice groups. CHIKV infected mice showed 100% mortality whereas treated mice (infected with DRDE 07) that received CHIK-VLPs IgG and then infected with CHIKV showed 100% survival rate same as mock infected mice that neither infected with CHIKV nor received specific IgG. However, treated mice (infected with DRDE 06), showed 90% survival. (B) Body weight gain measured on 1–7 day of post infection. Treated mice group (infected with DRDE 07 or DRDE 06) showed significantly higher (***P < 0.0001) body weight gain than CHIKV infected mice group; (C) Serum viremia at 3 dpi and 6 dpi. Serum viremia was found to be 10 fold lower at day 3 dpi in IgG treated groups (infected with DRDE 07 or DRDE 06) compared to CHIKV infected group (*P < 0.01). At day 6 dpi, 2500 fold lower CHIKV RNA copies were detected in treated groups (infected with DRDE 07 or DRDE 06) compared to CHIKV infected group (**P < 0.001).

To evaluate protective efficacy of CHIK-VLPs, *in vivo* neutralization was carried out in suckling mice model. Antibody raised against CHIK-VLPs were found to neutralize both the CHIKV strains (DRDE 07 and DRDE 06) significantly (**P < 0.001). Major clinical symptoms associated with Chikungunya in mice like hind limb paralysis and retarded growth was not discernable in CHIK-VLPs IgG treated mice group. Whereas, mice group that received control IgG and infected with CHIKV exhibited the typical clinical symptoms at 4–7 days of post infection. All the mice from CHIKV infected group died up to 7 days of post infection, whereas all mice from treated group survived and were healthy (Fig 7A). Body weight of mice, which had received passive immunization was similar to mock infected mice. In contrast, the mice revealed significantly higher body weight gain compared to CHIKV infected mice (***P < 0.0001) (Fig 7B). Serum viremia was found to be 10 fold lower at day 3 pi in IgG treated groups compared to CHIKV infected group (*P < 0.01). At day 6 pi, CHIKV RNA copies were found to be 2500 fold lower in treated groups compared to CHIKV infected group (**P < 0.001) (Fig 7C).

### Balance Th1/Th2 response has been observed by cytokine profiling

**Fig 8:**
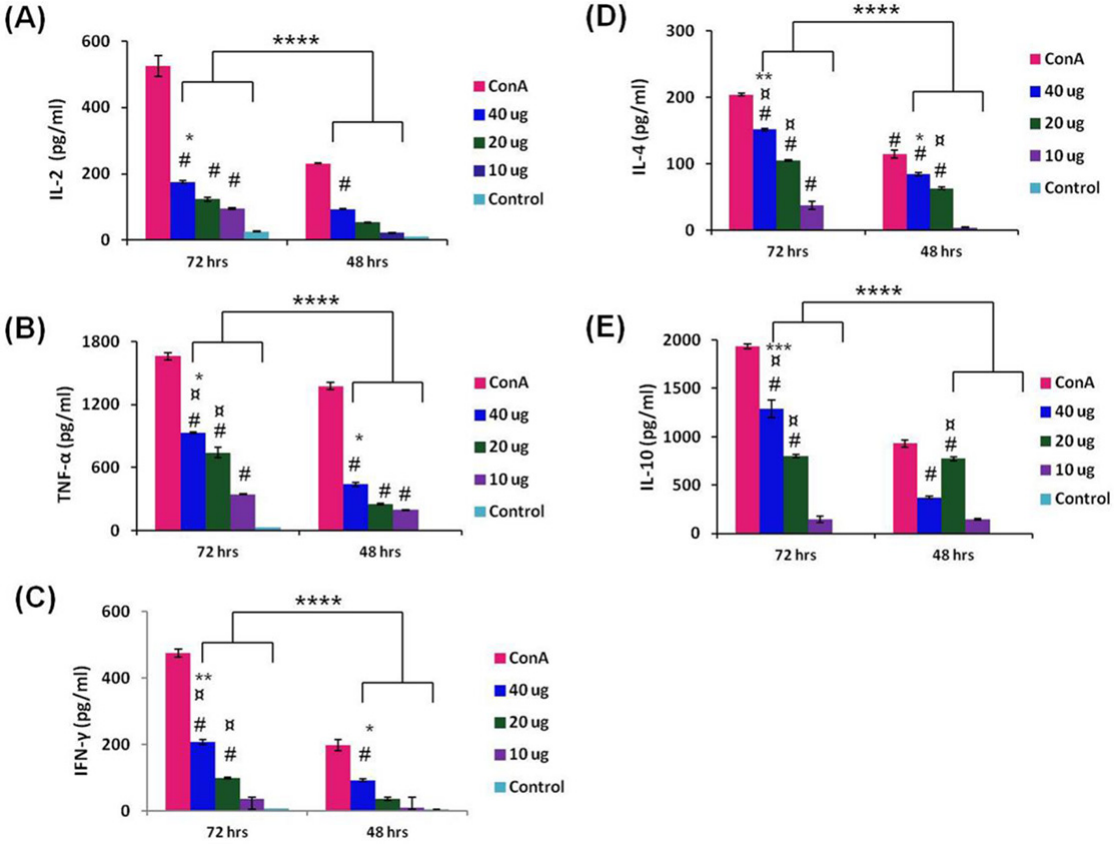
Measurement of cytokines expression in immunized mice splenocytes. Graphs showing concentrations of IL-2 **(A);** TNF-α **(B);** IFN-γ **(C)**; IL-4 **(D)** and IL-10 **(E)** in picograms per millilitre. Each bar represents the average of 10 mice/group ± S.D. and is representative of three independent experiments. Analysis was done by one way ANOVA, (Fisher LSD Method). ****P < 0.001 (significance between 72 and 48 hrs); ^**#**^P < 0.001(significance with respect to control); ^**¤**^P < 0.001(significance with respect to 10 μg inactivated CHIKV); *P < 0.05, **P < 0.01, ***P < 0.001(significance with respect to 20 μg inactivated CHIKV).

The expression level of IL-2, TNF-α, IFN-γ, IL-4 and IL-10 were seen to be significantly higher in all the immunized animal groups compared to control group (^**#**^P < 0.001). Expression level of IL-2, TNF-α, IFN-γ, IL-4 and IL-10 was significantly higher at 72 hrs, compared to 48 hrs among all the groups (****P < 0.001). A dose dependent pattern has also been seen in all cytokines. The expression of TNF-α, IFN-γ, IL-4 and IL-10 in 40 μg CHIK-VLPs was significantly higher compared with 20 μg (*P < 0.05, **P < 0.01, ***P < 0.001) and 10 μg CHIK-VLPs (^¤^P < 0.001) at 72 hrs (Fig 8B, 8C, 8D and 8E). The relative expression level of different cytokines in the immunized animal groups is listed in Table 1.

**Table 1:** Expression level of cytokines in control (PBS immunized) and vaccinated (CHIK-VLPs immunized) animal groups, stimulated with inactivated CHIKV.

S.No.	Groups	IL-2 (pg/ml)	TNF-α (pg/ml)	IFN-γ (pg/ml)	IL-4 (pg/ml)	IL-10 (pg/ml)
1.	40 μg CHIK-VLPs	175.49±3.41	932.50±7.00	207.65±6.47	151.54±1.89	1289.47±88.75
2.	20 μg CHIK-VLPs	123.78±6.18	741.23±48.09	98.12±2.27	104.98±1.55	798.48±18.29
3.	10 μg CHIK-VLPs	94.01±2.32	345.61±3.80	34.66±1.16	37.18±5.49	147.42±30.78
4.	Control	25.68±1.01	29.36±8.55	7.27±0.37	0.04±0.02	7.63±2.57

## Discussion

Chikungunya fever represents one of the classic examples of arboviral infection that has spread in most parts of the world over last decade. Inspite of its global presence, no licensed vaccine or antivirals are currently available. An effective vaccine has the potential to prevent human infection as well as stop the transmission of CHIKV. Though research on development of a CHIK vaccine has been initiated since mid 1980’s, however none was yet licensed.

A large number of heterologous proteins have been expressed either intracellular or in secreted form using *P*. *pastoris* system. There are a limited number of vectors for *P*. *pastoris* but most of them incorporated with tightly regulated and very strong AOX promoter [34, 35]. Here, pPIC9K vector was used, which is known to generate multicopy integrants and allows expression of recombinant proteins in secretory form using α-factor secretion signals. This α-factor secretion signals has a protease cleavage site that provides signals for secreted expression. Since native proteins of *P*. *pastoris* are secreted in very small amount, therefore, the secreted heterologous protein mainly comprises the total protein content of the culture medium [35, 36].

Recombinant *Pichia* clones have been generated with multi copy integrants, which harbor multiple copies of structural polyprotein gene of CHIKV. This is achieved through integration of transgene containing CHIKV structural polyprotein within *Pichia* genome via homologous recombination at multiple sites. This maintains stability of the target gene, over several passages. Further introduction of a mutation in one copy of the expression cassette has no effect on the total amount of expressed proteins as other copies resulting from multiple integration process contribute markedly in the compensation process [28, 37]. The host endopeptidase enables the separation of secretory signal from the expressed protein, resulting in the release of the structural polyprotein which assembled to form CHIK-VLPs [28, 30, 37].

Further the optimization of expression parameters of CHIK-VLP revealed YPD with 2% methanol as induction medium and yield of expressed protein was highest at 48 hrs. Induction of CHIK-VLPs expression was carried out using methanol which is very cheap and economical carbon source. In denaturing PAGE, approximately 52 KD, 35 KD and 13 KD bands have been observed which corresponds to the size of CHIKV E1/E2, capsid and E3 protein respectively. So, these observations indicated the presence of all structural proteins in the VLPs. The yield of CHIK-VLP was found to be 60 mg/l in this study. This further indicates the higher productivity of VLP in *Pichia* system compared to other systems like mammalian (10–20 mg/l) and insect expression (40 mg/l) [19]. These VLPs were successfully characterized by using different anti CHIKV specific antibodies via immunoblotting. The TEM results further indicated the morphological similarity of the CHIK-VLPs to the native CHIKV particles. Average diameter of Chikungunya cores was found approximately 40 nm which is consistent with the size of CHIKV and other alphaviruses. Thus, this observation is in agreement with the previous study [18, 19].

VLPs have been known to stimulate similar immune response as native virus. Polyvalence of VLPs leads to high frequency of cross linking between B-cell receptors and VLPs epitopes that resulted in stronger humoral response as well as broader protection [38]. VLPs vaccines against hepatitis B virus and human Papilloma virus are commercially available. These VLPs have served as polyvalent antigen and elicited prophylactic activity for human beings [24, 39, 40]. Therefore, *Pichia* system proved economical for large scale production of recombinant proteins with high safety [34]. This study also indicates that *P*. *pastoris* can serve as an alternate host system for large scale production of Chikungunya VLPs.

VLPs stimulate innate as well as adaptive immune response and act as safer effective vaccine candidate. Recently, phase I clinical trial on HEK cell based CHIK-VLPs has been successfully conducted by National Institute of Health (NIH) and it has been shown to be safe, well tolerated and immunogenic in healthy human volunteers [41].

Our evaluation indicated that the CHIK-VLPs elicited both humoral as well as cell mediated immune response in mice. The humoral immune response was characterized by high titers of ELISA antibodies. Further, high titer of neutralizing antibodies was demonstrated by plaque reduction neutralization assay. All doses exhibited promising antibody titre. The peak ELISA antibody titer was obtained on 42 days of post-vaccination; antibody titers were 3.2 × 10^4^, 2.56 × 10^4^ and 8 × 10^3^ with 40 μg, 20 μg and 10 μg CHIK-VLPs respectively. Humoral response plays a crucial role in neutralization of viruses and their clearance. In the present study, successful recognition of native CHIKV by antibodies raised against different doses of CHIK-VLPs was demonstrated in infected Vero cells. Virus neutralizing titre was determined for sera raised against different doses of CHIK-VLPs with two different viral strains belonging to ECSA genotype. The PRNT_50_ titer of mice sera was found to be dose dependent. This result support that antibodies raised against CHIK-VLPs not only effectively recognizes native CHIKV but also neutralize different strains of CHIKV. *In vivo* neutralization of different strains of CHIKV by anti CHIK-VLP sera was demonstrated in suckling mice through passive immunization. It was observed that CHIKV infection in suckling mice led to appearance of classic symptoms including increased viremia, retarded growth and hind limb paralysis. Immunized suckling mice were found to be protected as there was a 10 fold lower CHKV RNA copies at day 3 dpi and further 2500 fold lower CHIKV RNA copies were detected at day 6 dpi in IgG treated groups compared to virus control group. This passive immunization study clearly confirmed the ability of anti CHIK-VLP in both *in vivo* recognition as well as binding to antigenic sites of CHIKV leading to efficient neutralization.

Earlier studies of CHIK-VLPs in non human primates and mice revealed the important role of neutralizing antibody in the protection [18, 42]. In this study VLPs are shown to elicit high titer of neutralizing antibodies leading to protection against CHIKV in neonatal mice. The VLPs have also been recently shown to be more immunogenic compared to subunit antigens E1 or E2 in mouse model due to presence of higher density of antigenic epitopes [43].

Titre of IgG1 and IgG2a was comparable in CHIK-VLPs and inactivated CHIKV immunized mice. This demonstrates the effectiveness of CHIK-VLPs to induce a strong immune response comparable to inactivated CHIKV. In previous studies CHIK-VLPs have been shown to protect mice against CHIKV [18]. In these studies two booster doses with 19 μg CHIK-VLPs with adjuvant were required to protect mice, while in our study only single booster dose of even 10 μg CHIK-VLPs with adjuvant is sufficient to protect mice from CHIKV infection.

CHIKV specific cell mediated immune response against different formulations of CHIK-VLPs were evaluated through stimulation of splenocytes by inactivated CHIKV. Efficient stimulation of both humoral and cell mediated immune response is considered crucial for an effective vaccine. Hence the present study demonstrating induction of efficient cell mediated immune response by CHIK-VLPs is an important finding. Results of cell mediated response against CHIK-VLPs demonstrated the balanced Th1 and Th2 response. IL-2, TNF-α and IFN-γ mediate Th1 response and IL-4 and IL-10 mediate Th2 response. Higher expression of TNF-α, IL-10 and moderate expression of IL-2, IL-4 and IFN-γ indicated a balanced Th1/Th2 response. Significant up regulation of TNF-α, IL-2 and IgG2a favored the Th1 response whereas production of IL-4, IL-10 and IgG1 favored the Th2 response.

Role of cell mediated immune response in protection against CHIKV has been studied earlier in wild-type mice [44], where elevated level of TNF-α and IFN-γ helps in protection against natural CHIKV infection [45]. The induction of higher level of TNF-α and IFN-γ has been observed in mice immunized with different doses of CHIK-VLPs supports efficient generation of cell mediate immune response similar to that of native CHIKV response.

### Conclusion

In conclusion, this is the first report where, the potential of *P*. *pastoris* to express CHIK-VLPs has been demonstrated. CHIK-VLPs are also found immunogenic in mice and found efficient in inducing virus neutralizing antibodies and balance Th1/Th2 response. Purified IgG from CHIK-VLPs immunized mice sera showed protection against CHIKV viremia and hind limb paralysis in neonatal mice. These results confirm the protective efficacy of CHIK-VLPs against emerging Chikungunya virus.

## Supporting Information

S1 Fig Cloning of structural gene of Chikungunya virus in pPIC9K yeast transfer vector. (A) RT-PCR of PCR CHIKV structural polyprotein gene as Right and Left fragment; Lane M: GeneRuler 1 kb DNA Ladder (Fermentas, USA); Lane 1–4: Amplified Right fragment of CHIKV structural polyprotein gene; Lane 5–8: Amplified Left fragment of CHIKV structural polyprotein gene; (B) PCR analysis of pPIC9K-CHIKV-C-E3-E2-6K-E1 clone; M- DNA ladder, 1- PCR amplification from pPIC9K plasmid (-ve control), 2- PCR amplification from pPIC9K-CHIKV-C-E3-E2-6K-E1 clone, 3- PCR amplification from pTZ57-C/E3/E2/6K/E1(+ve control); (C) Restriction analysis of recombinant clone; M- DNA ladder, 1- pPIC9K-CHIKV-C-E3-E2-6K-E1 clone *SnaB*I & *Avr*II digest, 2- pPICK9K plasmid control *SnaB*I & *Avr*II digest, *3-* pPICK9K plasmid uncut; (D) Schematic diagram showing the CHIKV C-E3-E2-6K-E1expression within pPIC9K yeast transfer vector. The structural polyprotein gene of CHIKV is inserted at *Avr* II-*SnaB* I sites under the control of AOX1 promoter in fusion with the secretory signal (SS) at 5’ end. The transgene is integrated within the *Pichia* genome is at HIS 4 locus. Kan R gene present within the expression cassette confers resistance to Geneticin (in Yeast) and Kanamycin (in bacteria).
(TIF)

S2 Fig Screening of positive transformants. (A) Geneticin sensitivity assay for recombinant *P*. *pastoris* having structural polyprotein gene of Chikungunya virus integrated in genomic DNA.; (B) Genomic DNA PCR confirmation of transgene integration in CHIK-VLP*-Pichia* transformants; Lane M- DNA ladder (1 Kb), Lane 1–10 PCR amplification from Genomic DNA, Lane 11- PCR amplification from pPIC9K-CHIKV-C-E3-E2-6K-E1 plasmid DNA (Positive control), Lane 12- NTC.
(TIF)

S3 Fig Measurement of serum IgG isotypes titers in BALB/c mice immunized with inactivated CHIKV. Profile of IgG isotypes in sera after immunization with inactivated CHIKV (40 μg, 20 μg and 10 μg). Data represented in mean antibody titers with S.D. of ten Balb/c mice in each group. Analysis was done by one way ANOVA, (Fisher LSD) ^**#**^P < 0.0001(significance with respect to control); ****P < 0.0001(significance with respect to 20 μg inactivated CHIKV); °P < 0.0001(significance with respect to 10 μg inactivated CHIKV); ^**$**^P < 0.0001(significance with respect to IgG2b); ^§^P < 0.001(significance with respect to IgG2b); ^**¤**^P < 0.0001(significance with respect to IgG3).
(TIF)
