# Supplementary figures and images for: Expression and Characterization of Yeast Derived Chikungunya Virus Like Particles (CHIK-VLPs) and Its Evaluation as a Potential Vaccine Candidate
Source: PLoS Negl Trop Dis. 2016 Jul 11;10(7):e0004782. doi: 10.1371/journal.pntd.0004782 (PMC4939942; doi:10.1371/journal.pntd.0004782)

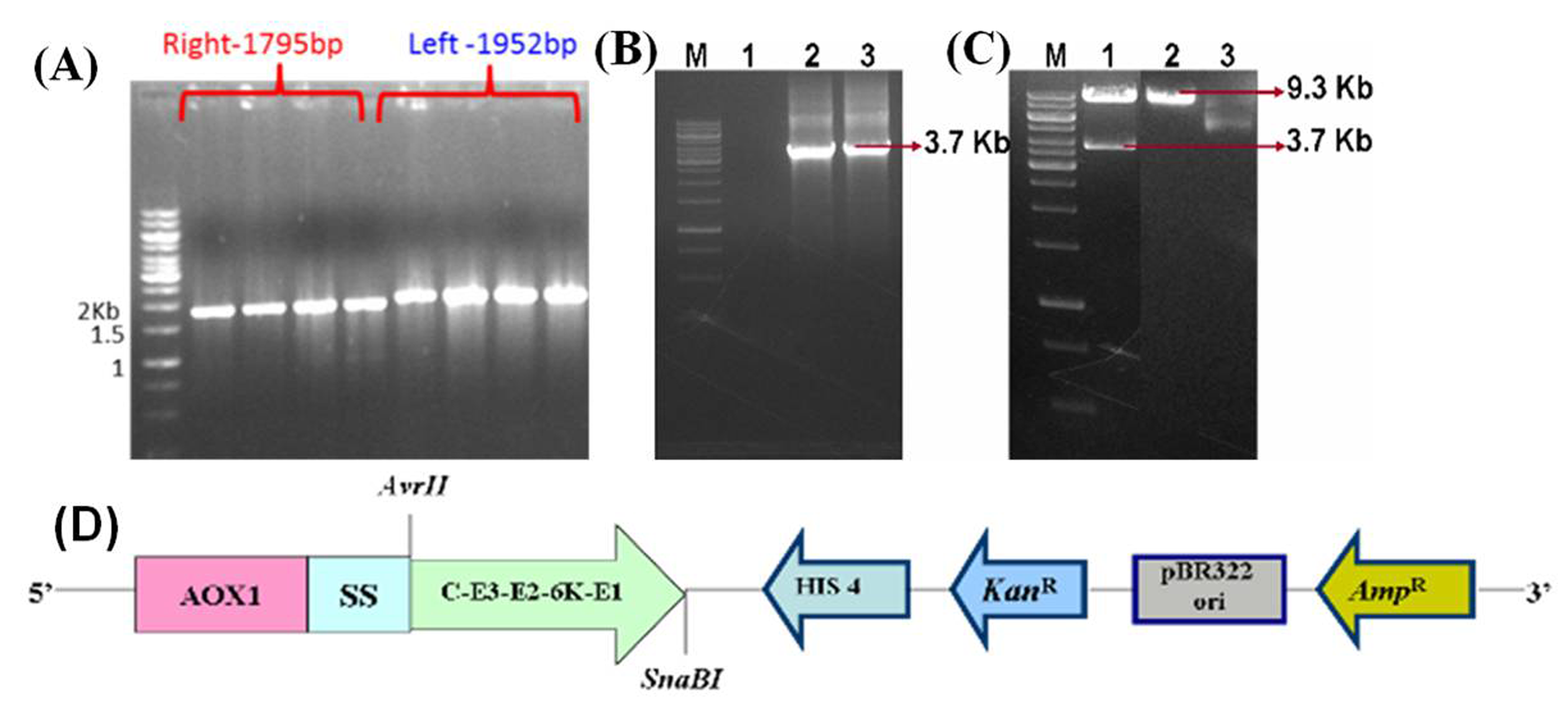

Supplement: S1 Fig — (A) RT-PCR of PCR CHIKV structural polyprotein gene as Right and Left fragment; Lane M: GeneRuler 1 kb DNA Ladder (Fermentas, USA); Lane 1–4: Amplified Right fragment of CHIKV structural polyprotein gene; Lane 5–8: Amplified Left fragment of CHIKV structural polyprotein gene; (B) PCR analysis of pPIC9K-CHIKV-C-E3-E2-6K-E1 clone; M- DNA ladder, 1- PCR amplification from pPIC9K plasmid (-ve control), 2- PCR amplification from pPIC9K-CHIKV-C-E3-E2-6K-E1 clone, 3- PCR amplification from pTZ57-C/E3/E2/6K/E1(+ve control); (C) Restriction analysis of recombinant clone; M- DNA ladder, 1- pPIC9K-CHIKV-C-E3-E2-6K-E1 clone SnaBI & AvrII digest, 2- pPICK9K plasmid control SnaBI & AvrII digest, 3- pPICK9K plasmid uncut; (D) Schematic diagram showing the CHIKV C-E3-E2-6K-E1expression within pPIC9K yeast transfer vector. The structural polyprotein gene of CHIKV is inserted at Avr II-SnaB I sites under the control of AOX1 promoter in fusion with the secretory signal (SS) at 5’ end. The transgene is integrated within the Pichia genome is at HIS 4 locus. Kan R gene present within the expression cassette confers resistance to Geneticin (in Yeast) and Kanamycin (in bacteria). (TIF) [file pntd.0004782.s001.tif]

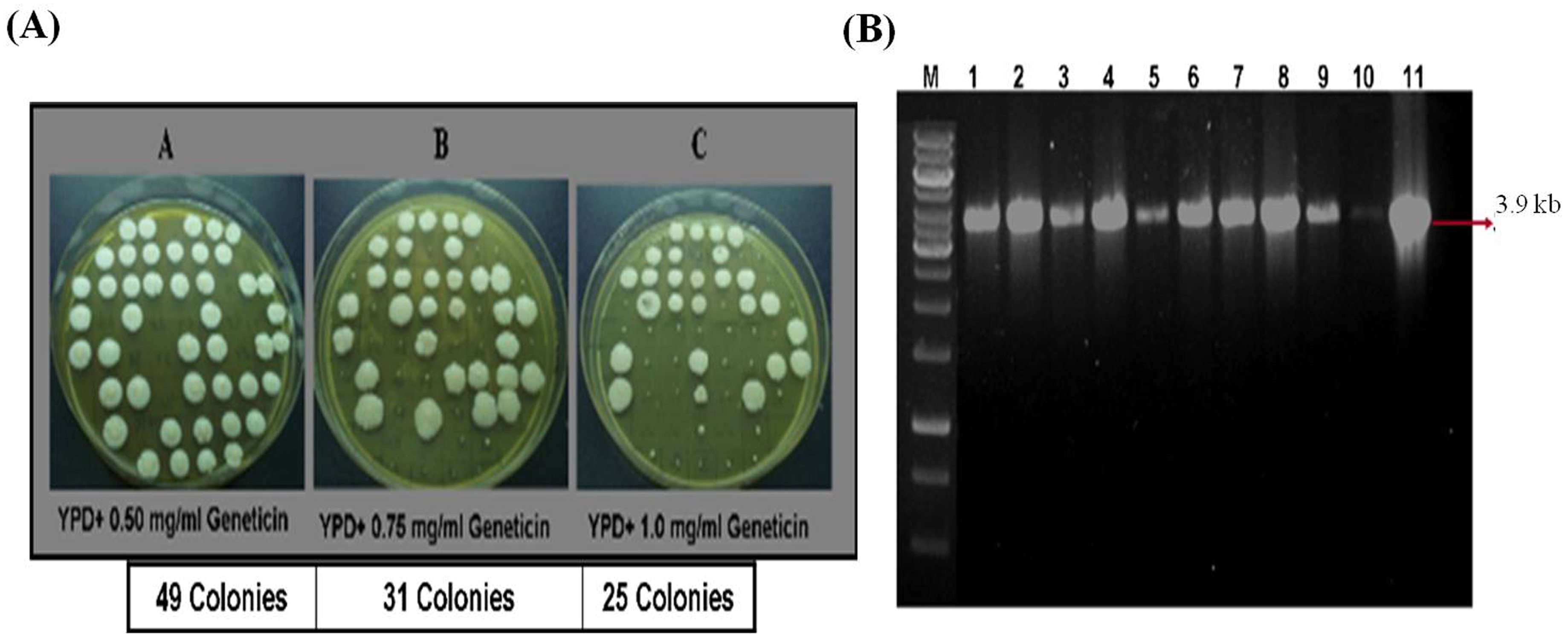

Supplement: S2 Fig — (A) Geneticin sensitivity assay for recombinant P. pastoris having structural polyprotein gene of Chikungunya virus integrated in genomic DNA.; (B) Genomic DNA PCR confirmation of transgene integration in CHIK-VLP-Pichia transformants; Lane M- DNA ladder (1 Kb), Lane 1–10 PCR amplification from Genomic DNA, Lane 11- PCR amplification from pPIC9K-CHIKV-C-E3-E2-6K-E1 plasmid DNA (Positive control), Lane 12- NTC. (TIF) [file pntd.0004782.s002.tif]

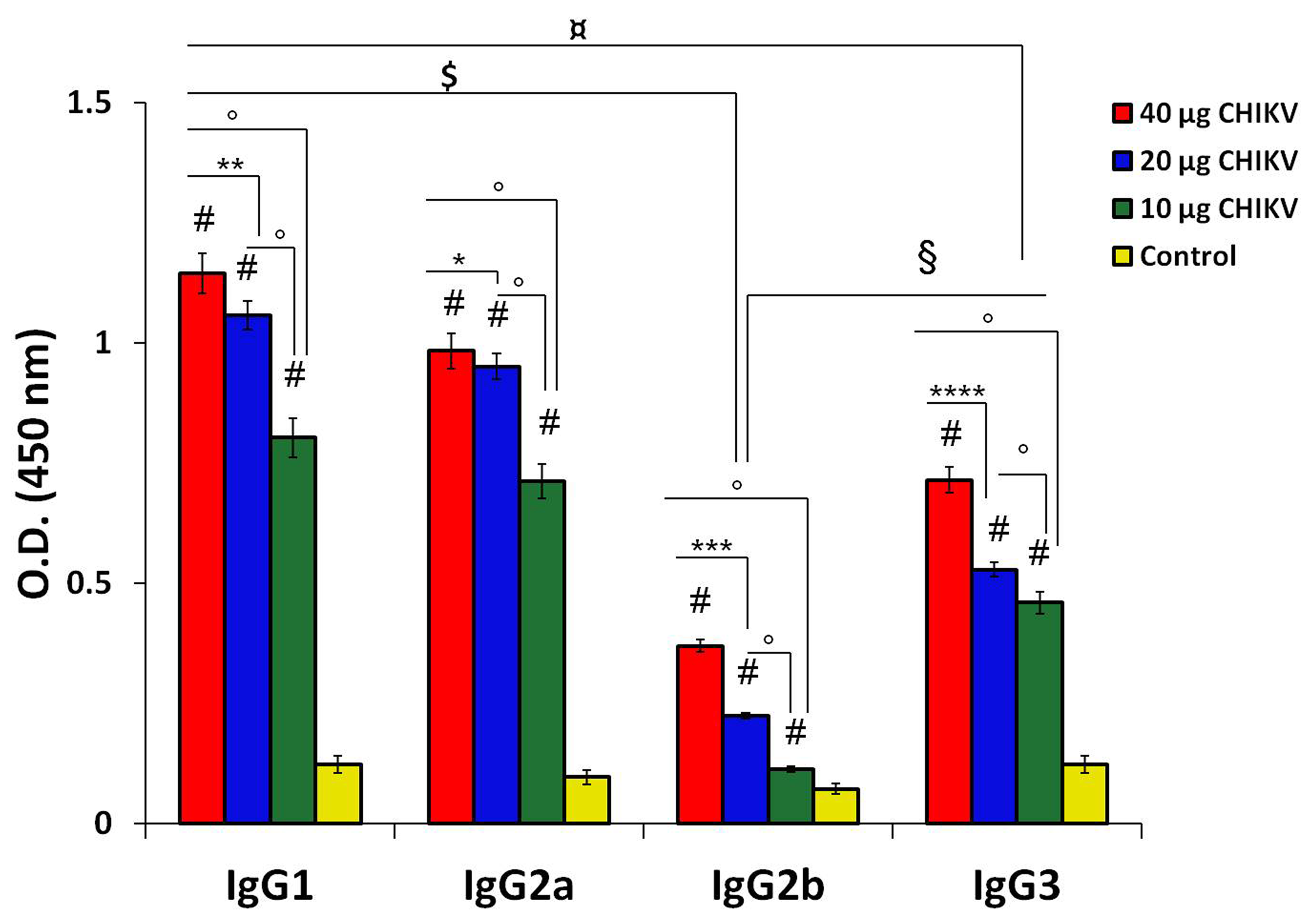

Supplement: S3 Fig — Profile of IgG isotypes in sera after immunization with inactivated CHIKV (40 μg, 20 μg and 10 μg). Data represented in mean antibody titers with S.D. of ten Balb/c mice in each group. Analysis was done by one way ANOVA, (Fisher LSD) #P < 0.0001(significance with respect to control); ****P < 0.0001(significance with respect to 20 μg inactivated CHIKV); °P < 0.0001(significance with respect to 10 μg inactivated CHIKV); $P < 0.0001(significance with respect to IgG2b); §P < 0.001(significance with respect to IgG2b); ¤P < 0.0001(significance with respect to IgG3). (TIF) [file pntd.0004782.s003.tif]
